# Author Correction: Clinically-relevant postzygotic mosaicism in parents and children with developmental disorders in trio exome sequencing data

**DOI:** 10.1038/s41467-022-33386-7

**Published:** 2022-09-27

**Authors:** C. F. Wright, E. Prigmore, D. Rajan, J. Handsaker, J. McRae, J. Kaplanis, T. W. Fitzgerald, D. R. FitzPatrick, H. V. Firth, M. E. Hurles

**Affiliations:** 1 Institute of Biomedicine and Clinical Science, College of Medicine and Health, University of Exeter, RILD Building, Royal Devon and Exeter Hospital, Exeter, EX2 5DW UK; 2 Wellcome Sanger Institute, Wellcome Genome Campus, Hinxton, Cambridge, CB10 1SA UK; 3 European Bioinformatics Institute (EMBL-EBI), Wellcome Genome Campus, Cambridge, CB10 1SD UK; 4 MRC Human Genetics Unit, University of Edinburgh, Edinburgh, EH4 2XU UK; 5 Clinical Genetics, Box 134 Addenbrooke’s Hospital, Cambridge University Hospitals, Cambridge, UK

**Correction to**: *Nature Communications* 10.1038/s41467-019-11059-2, published online 5 July 2019

The original version of the Supplementary Information associated with this Article included an incorrect Supplementary Data 2 file, in which Supplementary Data 1 was duplicated. The HTML has been updated to include a corrected version of Supplementary Data 2.

## Supplementary information

Updated Supplementary Data 2

