# Supplementary material for: Clinically-relevant postzygotic mosaicism in parents and children with developmental disorders in trio exome sequencing data
Source: Nat Commun. 2019 Jul 5;10:2985. doi: 10.1038/s41467-019-11059-2 (PMC6611863; doi:10.1038/s41467-019-11059-2)
Supplement: Supplementary file 1 — Supplementary Information [file 41467_2019_11059_MOESM1_ESM.pdf]

Supplementary Figure 1. Characteristics of child-PZM

(a)

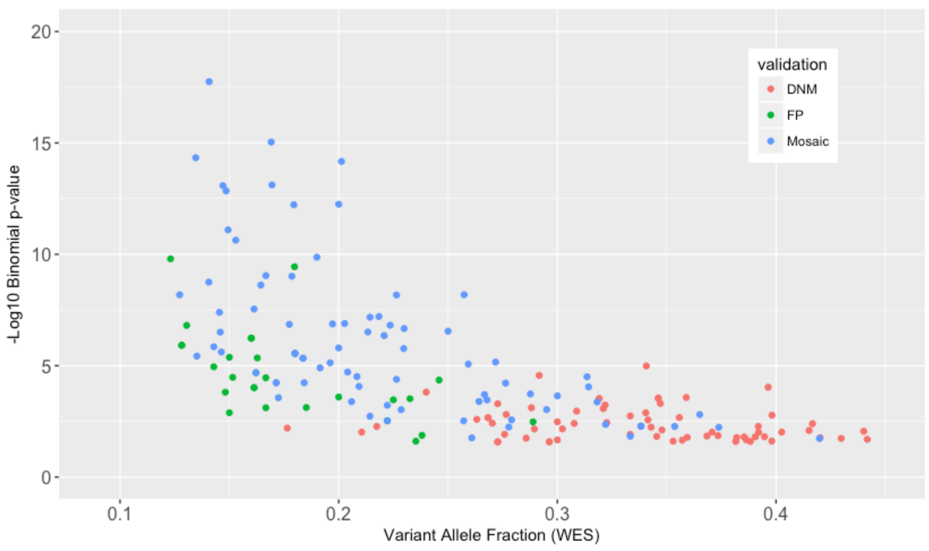

(b)

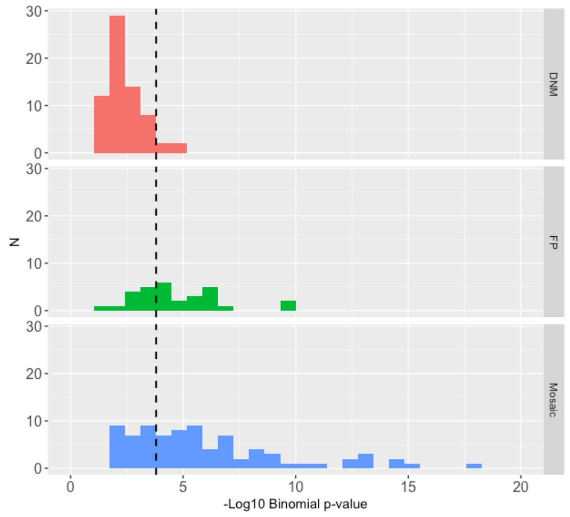

(c)

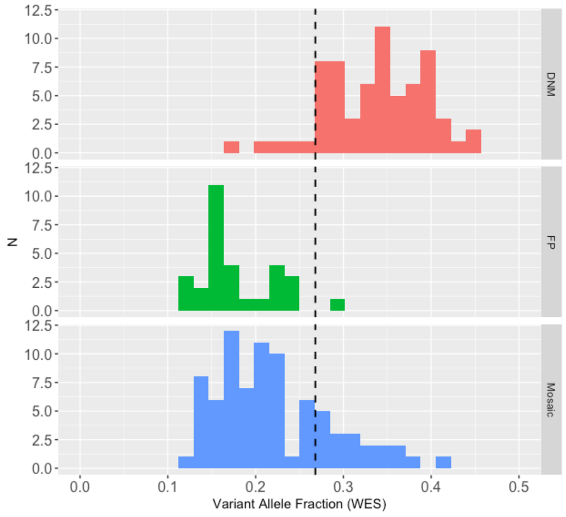

(d)

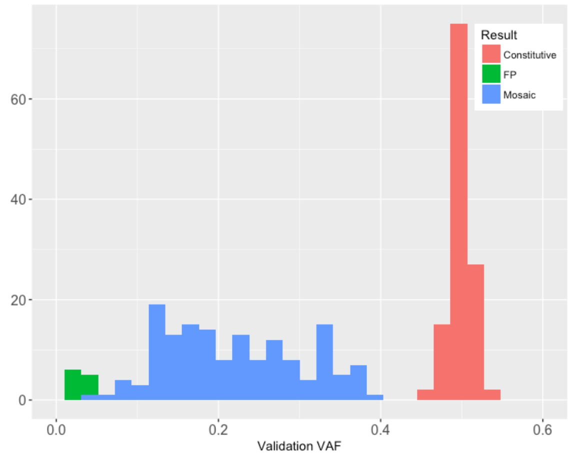

(e)

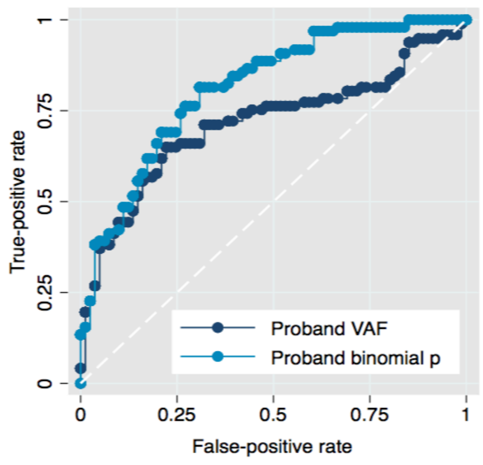

(a) Scatter plot of exome VAF for the child versus its binomial p-value (centred around 0.5), coloured by ultrahigh-depth sequencing validation result; **(b)** Histogram of proband WES VAF separated by validation result; **(c)** Histogram of proband WES binomial p-value separated by validation result; **(d)** Histogram of proband validation VAF separated by validation result; **(e)** ROC-curve for VAF (AUROC = 0.71) and binomial p-value (AUROC = 0.81) for a variant being mosaic versus not. Red = constitutive *de novo* mutation (DNM); blue = mosaic DNM (child-PZM); green = false positive (FP), i.e. variant not present; black dotted line = best threshold between mosaic and other validation results.

Supplementary Figure 2. Characteristics of low-level parent-PZM

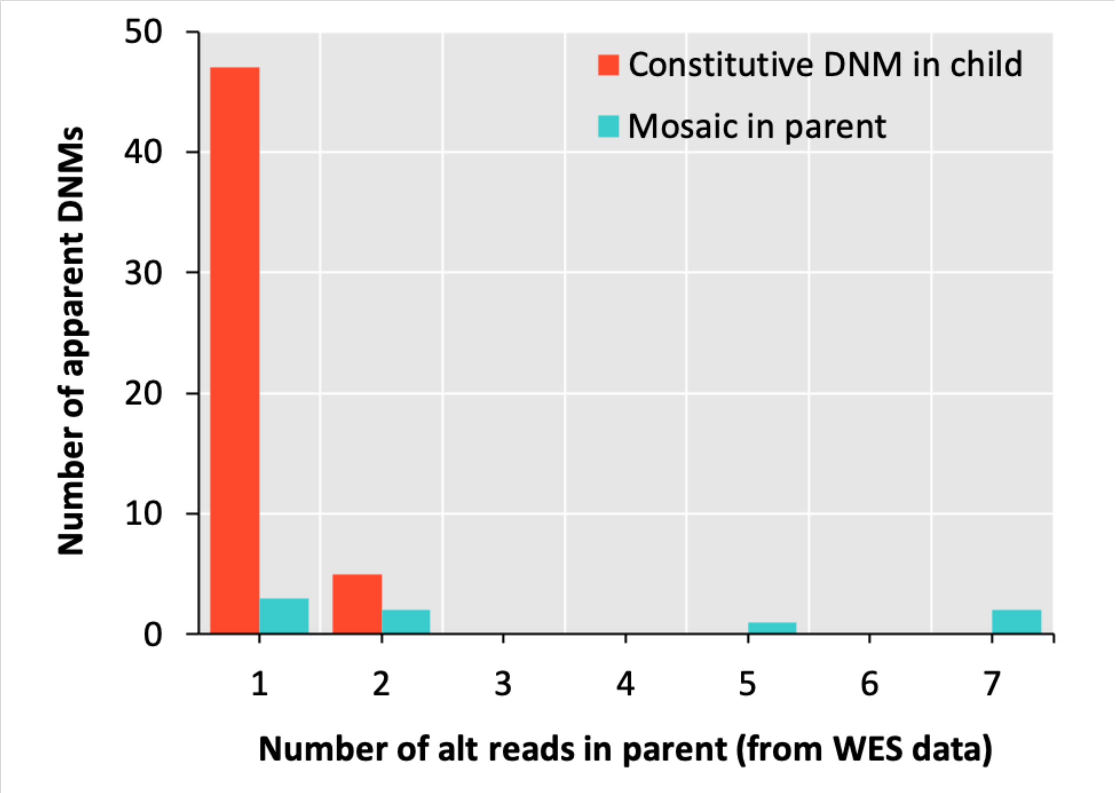

Histogram of number of alt reads in the parent for 72 apparent DNMs, separated by validation result. Red = constitutive DNM in child; aquamarine = mosaic in parent (Parent-PZM) and constitutive in child.

Supplementary Figure 3. VAF of candidate mosaic *de novo* and a nearby inherited variant on the same read-pair

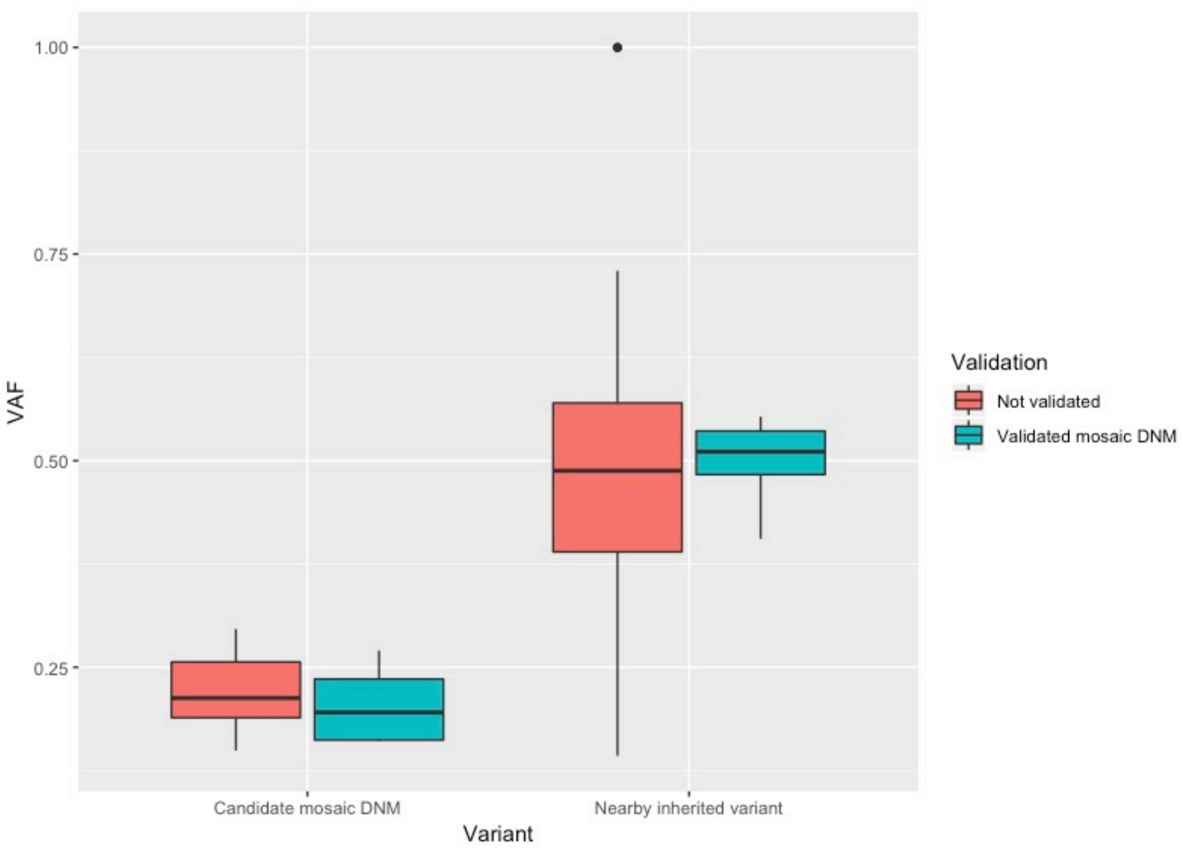

Boxplot of the exome VAF of 41 candidate mosaic variants (6 validated) compared with the VAF of a nearby informative inherited variant on the same read-pair.

Supplementary Figure 4. Enrichment of *de novo* mutations with parental age

(a)

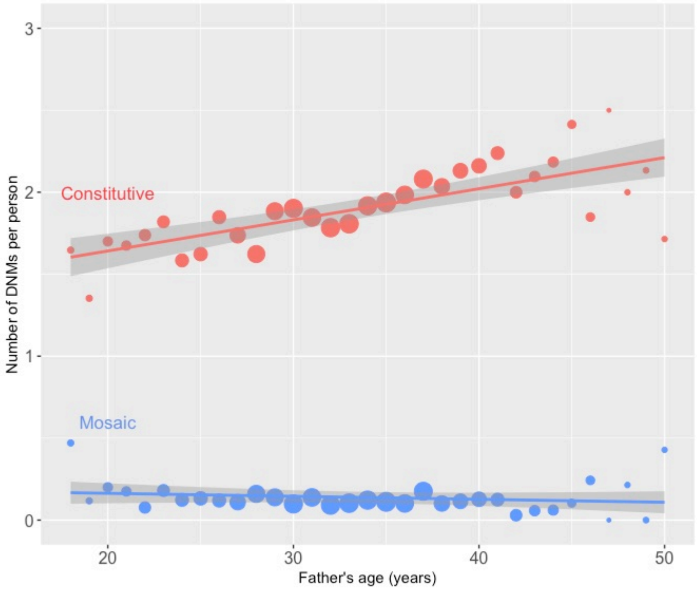

(b)

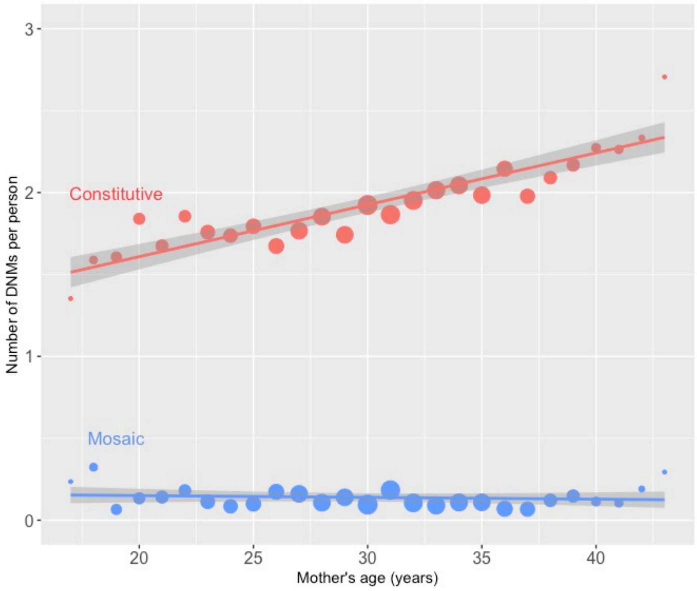

Relationship between **(a)** paternal and **(b)** maternal age and average number of high stringency *de novo* mutations (DNMs) per person, split between likely mosaic variants (variant allele fraction, VAF <0.27; blue) and likely constitutive variants (VAF>0.27; red). The linear regression coefficient for mosaic variants is not significantly different from the null in either plot (p>0.2). Point size represents the number of trios at each age, and grey zones represent 95% confidence intervals.

Supplementary Figure 5. Estimate of the proportion of mosaicism using WES data

(a)

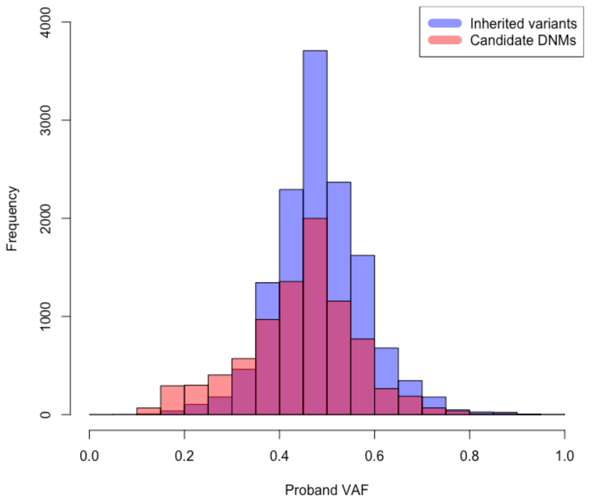

(b)

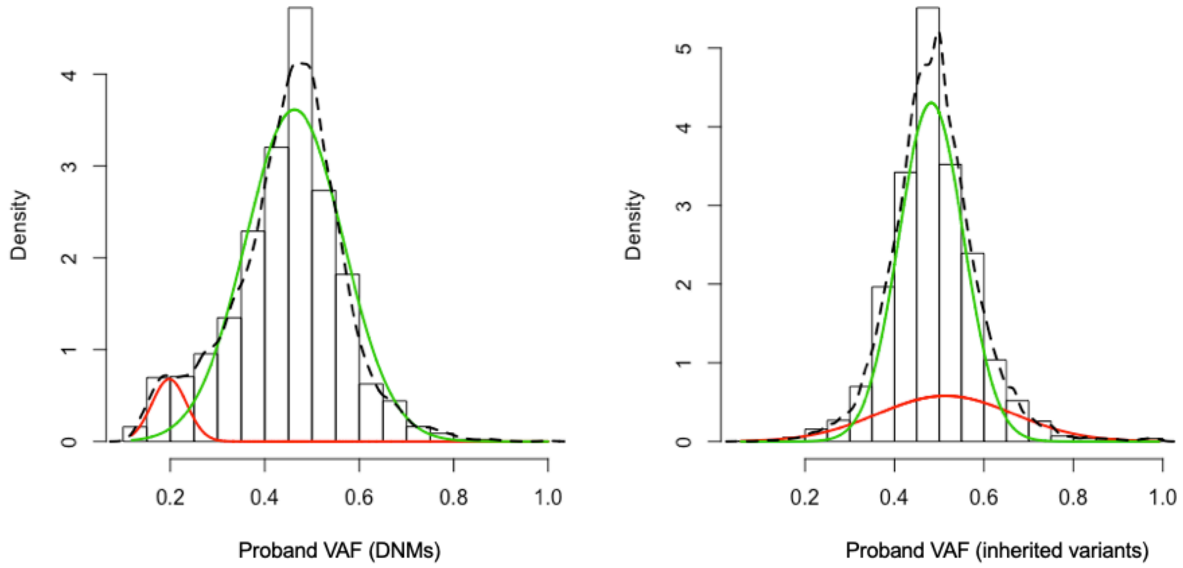

(a) Comparison of the VAF distribution of candidate DNMs (n=8,464) with our list of potentially pathogenic inherited variants (n=10,721), showing an excess of potentially mosaic variants in the former (red) that are not present in the latter (blue). **(b)** A Gaussian Mixture Model fitted to the distribution of VAFs from candidate DNMs and inherited variants using the R package *mixtool*s, with starting values of mean= 0.5 and component proportion=90% for constitutive variants (green), and mean=0.2 and component proportion=10% for mosaic variants of (red). Approximately ~6% of DNMs and 0% of the inherited variants were estimated to belong to a distinct mosaic population (mean VAF=0.198).

Supplementary Figure 6. Modelling of the probability of identifying child-PZM variants with different levels of mosaicism given different sequencing coverage

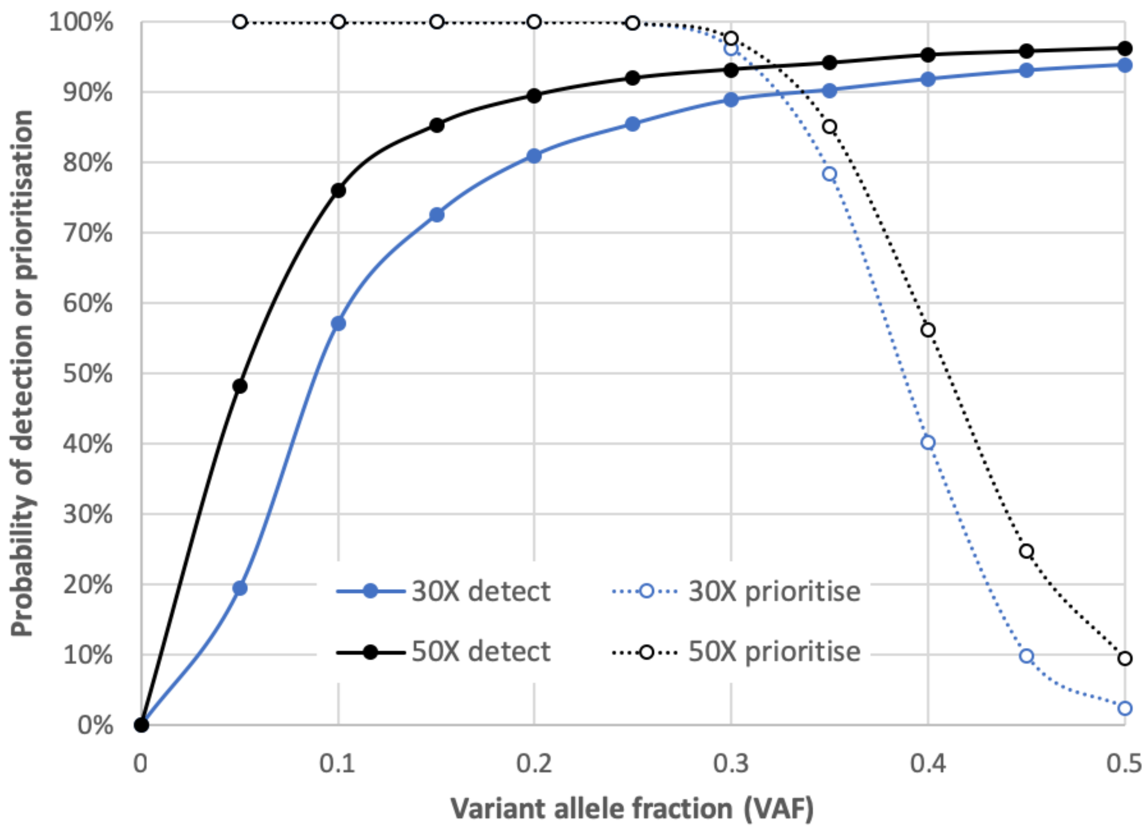

A binomial distribution was used to model the mean depth of coverage (30X and 50X) and mean VAF (0-0.5) then the number of ALT reads and binomial p-value were calculated for non-negative values. Since our DNM detection pipeline requires >2 ALT reads for a variant to be detected (>1 on the forward and >1 on the reverse strand), the probability (i.e. power) of detecting a variant is the proportion of trials in which ALT>2 (solid lines). Once detected, the probability of a variant being prioritised for validation as a candidate mosaic variant is the proportion of trials where the FDR<0.2 (dashed lines).
