# Supplementary material for: Clinically-relevant postzygotic mosaicism in parents and children with developmental disorders in trio exome sequencing data
Source: Nat Commun. 2019 Jul 5;10:2985. doi: 10.1038/s41467-019-11059-2 (PMC6611863; doi:10.1038/s41467-019-11059-2)
Supplement: Supplementary file 4 — Description of Additional Supplementary Files [file 41467_2019_11059_MOESM4_ESM.docx]

**Title:** Supplementary Data 1.
**Description:** List of validated pathogenic and likely pathogenic mosaic variants in 4,293 DDD trios

**Title:** Supplementary Data 2.
**Description:** List of all validated mosaic variants
